# Associations between daily neighborhood crime and physical disorder exposure and sleep patterns in adolescents

**DOI:** 10.1093/sleep/zsag226

**Published:** 2026-08-19

**Authors:** Stephanie L Mayne, Gabrielle DiFiore, Alexander G Fiks, Hannah Martin, Vicky Tam, Jonathan A Mitchell, Ian Barnett, Douglas J Wiebe, Mathias Basner, Karen Glanz

**Affiliations:** Clinical Futures, Policy Lab, and the Possibilities Project, Children’s Hospital of Philadelphia, Philadelphia, PA, United States; Department of Pediatrics, University of Pennsylvania, Perelman School of Medicine, Philadelphia, PA, United States; Leonard Davis Institute for Healthcare Economics, University of Pennsylvania, Perelman School of Medicine, Philadelphia, PA, United States; Clinical Futures, Policy Lab, and the Possibilities Project, Children’s Hospital of Philadelphia, Philadelphia, PA, United States; Clinical Futures, Policy Lab, and the Possibilities Project, Children’s Hospital of Philadelphia, Philadelphia, PA, United States; Department of Pediatrics, University of Pennsylvania, Perelman School of Medicine, Philadelphia, PA, United States; Leonard Davis Institute for Healthcare Economics, University of Pennsylvania, Perelman School of Medicine, Philadelphia, PA, United States; Clinical Futures, Policy Lab, and the Possibilities Project, Children’s Hospital of Philadelphia, Philadelphia, PA, United States; Data Science and Biostatistics Unit, Children’s Hospital of Philadelphia, Philadelphia, PA, United States; Department of Pediatrics, University of Pennsylvania, Perelman School of Medicine, Philadelphia, PA, United States; Division of Gastroenterology, Hepatology, and Nutrition, Children’s Hospital of Philadelphia, Philadelphia, PA, United States; Department of Biostatistics, Epidemiology, and Informatics, University of Pennsylvania, Perelman School of Medicine, Philadelphia, PA, United States; Department of Epidemiology, University of Michigan School of Public Health, Ann Arbor, MI, United States; Department of Psychiatry, University of Pennsylvania Perelman School of Medicine, Philadelphia, PA, United States; Leonard Davis Institute for Healthcare Economics, University of Pennsylvania, Perelman School of Medicine, Philadelphia, PA, United States; Department of Biostatistics, Epidemiology, and Informatics, University of Pennsylvania, Perelman School of Medicine, Philadelphia, PA, United States; Department of Biobehavioral Health Sciences, School of Nursing, University of Pennsylvania, Philadelphia, PA, United States

**Keywords:** sleep, crime, physical disorder, actigraphy, global positioning system

## Abstract

**Study Objectives:**

While growing evidence suggests neighborhood safety and physical disorder may impact adolescent sleep, prior research has not accounted for mobility outside the home neighborhood. This study examined associations of crime and physical disorder surrounding adolescents’ homes, schools, and in their daily activity spaces with sleep patterns.

**Materials and Methods:**

Adolescents aged 15–18 years (*n* = 142) were followed for 14 days using smartphone global position systems (GPS) tracking and actigraphy. GPS locations recorded during adolescents’ waking hours were spatially linked to local data on crime and vacant lots to derive exposures surrounding adolescents’ homes, schools, and daily activity spaces. Mixed-effects linear and modified Poisson models estimated associations of crime and vacant lots with sleep duration, timing, efficiency, insufficient (<8 hours) and very short sleep (<6 hours). Fixed-effects models estimated associations of within-person differences in activity-space crime and vacant lot exposures with sleep patterns.

**Results:**

Higher crime exposure surrounding adolescents’ homes and schools was associated with shorter sleep duration, higher risk of insufficient and very short sleep, earlier offset timing (home only) and later onset timing (school only). Higher activity-space exposure to vacant lots was associated with shorter sleep duration, later onset, and higher risk of insufficient sleep. On days when adolescents’ exposure to vacant lots was higher relative to their own average, they had shorter sleep duration, earlier offset timing, and higher risk of insufficient and very short sleep, while such associations were not seen for neighborhood crime.

**Conclusions:**

Associations of crime and vacant lot exposures with actigraphy-assessed adolescent sleep patterns varied by setting.

## Graphical Abstract

**Graphical Abstract:**
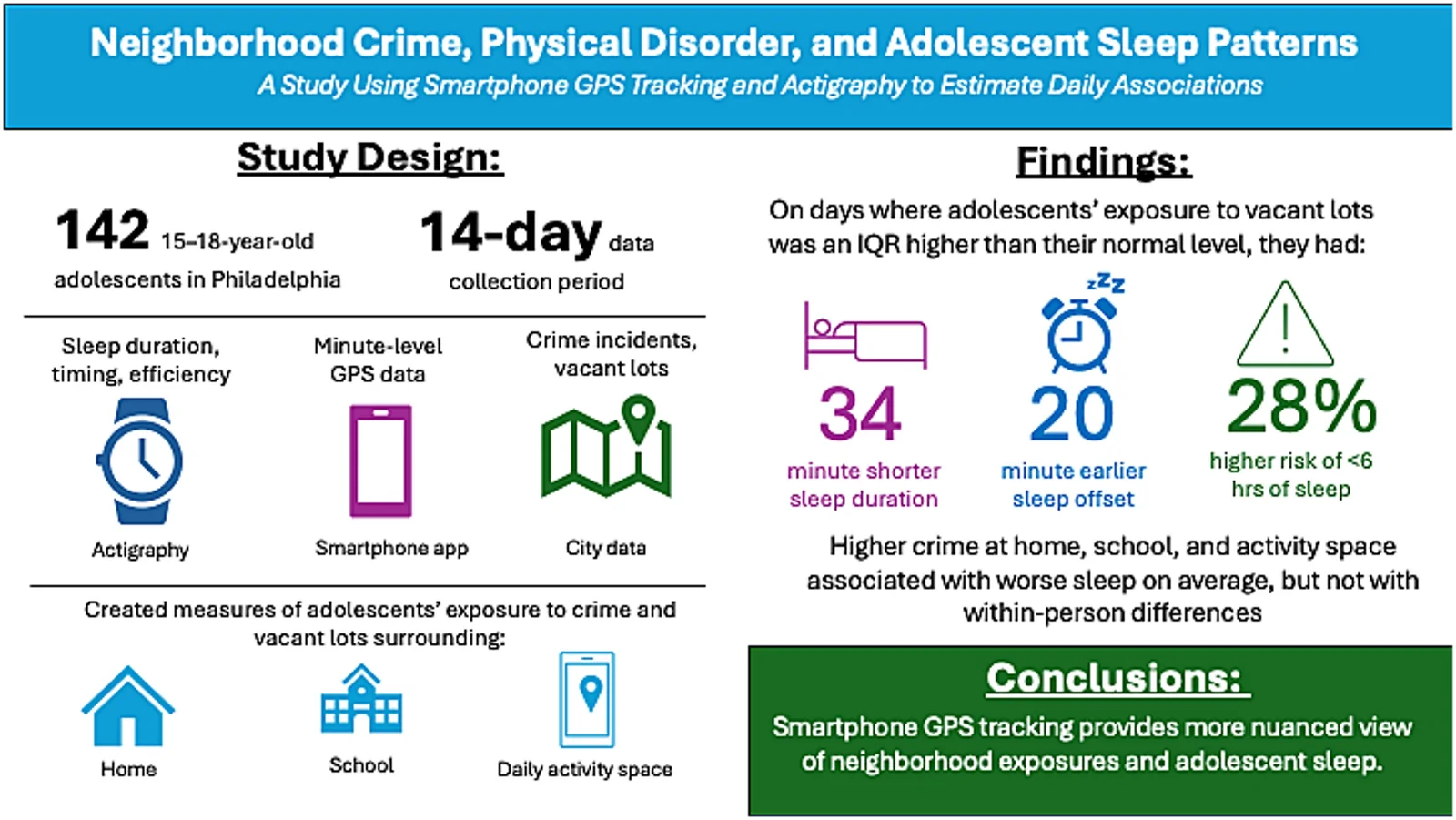

## Teaser

Neighborhood stressors like crime and physical disorder may adversely impact sleep among adolescents, but prior studies have not examined exposures that account for mobility outside the home. This study is the first to combine global positioning system location tracking and actigraphy to determine associations of home, school, and daily activity-space crime and vacant lot exposure with objectively-measured sleep duration, timing, and efficiency among adolescents. Higher home and school exposure to crime was associated with more adverse sleep outcomes, as was higher exposure to vacant lots in adolescents’ activity spaces. On days when adolescents’ exposure to vacant lots was higher than their average level, they had shorter sleep duration, earlier offset, and higher risk of insufficient sleep, while within-person changes in crime exposure were not associated with sleep outcomes. Results underscore the potential importance of quantifying environmental exposures throughout daily activities, beyond the residential neighborhood.

## Study Objectives

Over 75% of U.S. high school students do not sleep at least 8 hours per night as recommended by the American Academy of Sleep Medicine [1, 2]. While sleep is a complex behavior impacted by a multitude of biological and behavioral features, evidence is growing that environmental exposures may impact adolescent sleep, including features of the neighborhoods where adolescents live [3–7]. Due to structural and political factors including racial residential segregation and neighborhood disinvestment [8], individuals within the same city may experience very different neighborhood environments, with distinct environmental risks and supports.

Stressors in the neighborhood environment, including crime and physical disorder, are hypothesized to adversely impact sleep by increasing arousal, either directly or via stress [9], making it more difficult to fall and stay asleep. Among adolescents, lower perceived safety and greater community violence concerns have been associated with more adverse sleep patterns and self-reported sleep problems [4, 9–13], though not all studies have found associations [7]. Fewer studies have examined objective measures of neighborhood crime; however, two studies assessing daily crimes in adolescents’ home neighborhoods found that same-day crime exposure was associated with later sleep timing [14] and lower sleep efficiency [15]. A strength of these studies is the ability to examine within-person associations, which account for unmeasured confounding by factors that do not vary day to day [16].

Neighborhood physical disorder, which includes visible signs of deterioration or disinvestment such as vacant lots, trash, and graffiti, is associated with poorer mental health [17] and has been found to be associated with shorter sleep duration [18] and poorer sleep quality among adults [19, 20]. While neighborhood physical disorder is hypothesized to impact sleep through stress [21], its relationship with sleep outcomes among adolescents has been understudied. In the National Survey of Child Health, which included both children and adolescents, parents who rated their neighborhood as having garbage/litter, rundown housing, and signs of vandalism were more likely to report their child did not regularly get enough sleep [6]. In contrast, two studies using data from the Boston Youth Survey found no evidence of association between neighborhood disorder and self-reported sleep duration [22, 23]. Since neighborhood physical disorder is a modifiable characteristic that may be impacted by interventions such as vacant lot remediation [24], a better understanding of its relationship with adolescent sleep is needed to inform sleep promoting policies.

Although this is a growing area of research, understanding the relationship between neighborhood crime, physical disorder, and adolescent sleep is limited by the fact that prior studies have focused almost entirely on the residential neighborhood (e.g. the immediate neighborhood surrounding the home) [3]. Neighborhood exposures are typically identified by the administrative unit (e.g. census tract) in which study participants’ residence is located, or by aggregating exposures within a buffer (e.g. 1 km) around the home. However, adolescents may spend much of their day away from home for school and daily activities [25, 26], especially as they become older and gain additional independence [25]. As such, neighborhood environmental measures based on the home neighborhood in isolation may only partially explain adolescents’ interactions during daily activities with environmental exposures that are hypothesized to impact sleep. Consistent with this idea, our prior pilot study among adolescents in Philadelphia indicated that exposures to common environmental features, such as crime and greenspace, differed significantly between adolescents’ home neighborhoods and other locations where they spent time [27].

This limitation may be overcome by using global position systems (GPS) to track individuals’ locations through time and space. GPS coordinates may then be linked with data on neighborhood features using Geographic Information Systems and used to estimate environmental exposures, such as crime or physical disorder, within individuals’ activity spaces. “Activity space” is defined as the set of locations an individual encounters as they go about their daily lives [28]. Adolescents’ activity space-based exposures (or “activity-space exposures”) include not only the home neighborhood, but environmental context encountered at school, in travel, and in other locations of daily activities, which may improve exposure assessment and better explain the relationship of neighborhood environments with adolescent sleep. Although this approach has been used to examine associations of neighborhood crime [29] and walkability [30] with sleep in adults, and to examine outcomes such as adolescent substance use [31–34] and physical activity [35–37], to our knowledge prior studies have not examined the association of activity-space neighborhood environmental exposures with adolescent sleep. An additional benefit of GPS location tracking is the ability to assess environmental exposures that vary day-to-day, enabling examination of associations of within-person differences in environmental exposures and within-person differences in sleep outcomes across nights.

The objective of our study was to determine the association of home, school, and daily activity-space measures of crime and physical disorder with nightly sleep duration, timing, and efficiency among 15-to-18-year-old adolescents. We hypothesized that greater exposure to crime and physical disorder around adolescents’ homes, schools, and daily activity spaces would be associated with shorter sleep duration, later sleep onset timing, lower sleep efficiency, and higher risk of insufficient sleep. Leveraging 14 days of continuous GPS monitoring and actigraphy, we also examined within-person associations of daily activity-space exposures and differences in sleep patterns. We hypothesized that adolescents would have more adverse sleep patterns on days where their activity-space exposure to crime and vacant lots exceeded their average level.

## Materials and methods

### Study population

The Teen Neighborhood Activity Patterns and Sleep (Teen NAPS) study, conducted between February 2022 and August 2024, was a cross-sectional study that enrolled adolescents for a 14-day period using intensive longitudinal data collection [38]. Eligible participants were (1) aged 15–18 years old, (2) lived and attended school in Philadelphia, Pennsylvania, (3) spoke English, and (4) had a smartphone with a text/data plan. Adolescents with a health condition that might impact sleep or mobility, who used medications that might impact sleep, or who were experiencing an acute illness at the time of enrollment that might impact daily activity were excluded. Adolescents were recruited via email invitations to families in the Children’s Hospital of Philadelphia (CHOP)’s network with an adolescent in the eligible age range and an address in the city of Philadelphia, as well as via advertisements in CHOP primary care practices and on the main CHOP hospital campus. After completing a screening survey and informed consent/assent, participants completed baseline surveys using REDCap electronic data capture [39]. Upon baseline survey completion, adolescents were mailed an actigraphy device to measure their sleep patterns in their home setting. Upon receiving the actigraphy device, adolescents completed a virtual study visit via Health Insurance Portability and Accountability Act (HIPAA)-compliant video conferencing application where a study team member assisted them in initializing the actigraphy device and downloading a smartphone app for GPS location tracking. Each adolescent’s 14-day data collection period began the following day. The CHOP Institutional Review Board approved the study.

### Sleep assessment

To assess sensor-based measures of sleep duration and timing, we used wrist-worn GENEActiv Original devices (Activinsights Ltd.), which collect raw acceleration data using tri-axial accelerometry. The devices collected acceleration data at a sampling rate of 50 Hz. Adolescents pressed a button on the GENEActiv to initialize data collection during the virtual visit and were instructed to wear the device on their non-dominant wrist at all times for 14 days. Acceleration data were processed using the open source GGIR package [40, 41], which used algorithms to pre-process the data, identify non-wear periods, estimate sleep and wake, and detect sleep periods. For sleep–wake scoring, the Cole-Kripke sleep detection algorithm [42] within GGIR was selected, informed by a recent validation study that demonstrated that this sleep–wake scoring method has strong ability to detect sleep (sensitivity = 0.80) and wake (specificity = 0.79) in children when integrated into GGIR [43]. Adolescents were asked to report their sleep and wake times each morning on a prompt sent via a smartphone ecological momentary assessment app (LifeData, lifedatacorp.com). However, as in prior work [43], we used the automated sleep period detection method within GGIR (HDCZA) without including self-reported sleep diary data in order to avoid introducing recall bias and to minimize missing data, as 17.9% of nights lacked sleep diary data. Actigraphy data outside the 14-day observation period was excluded, as were nights with insufficient data quality as described in the Supplementary Methods. We classified nights as school (Sunday–Thursday) and non-school (Friday–Saturday) nights, with the night preceding federal holidays classified as non-school nights. Sleep outcomes were generated at the night level (up to 14 per participant) and included continuous duration (minutes per night, calculated as number of minutes of sleep between onset and offset), onset and offset timing (operationalized as minutes from midnight, e.g. -5.0 = 23:55, 60.0 = 01:00, based on the first and last sustained inactivity bout during the sleep period), and sleep efficiency (percentage of the sleep period classified as sleep). We also created dichotomous measures of insufficient sleep (sleep duration <8 hours) and very short sleep (sleep duration <6 hours).

### GPS location tracking

Location tracking was completed using Aware, an open-source toolkit for collecting contextual information from smartphones using built-in sensors [44]. Participants installed Aware on their personal smartphone and were asked to leave it passively running for the entire 14-day data collection period. The study-specific Aware app was programmed to passively collect location data using GPS, cell tower, and WiFi triangulation and transmit time-stamped latitude/longitude points, movement speed, and estimated location accuracy to a secure server every 60 seconds throughout the data collection period. GPS data were examined for inaccurate or implausible values [45], and days with <10 hours of valid GPS data were excluded [46] (Supplementary Methods). We classified each GPS location point as occurring during sleep or wake by comparing the GPS point’s timestamp to the sleep onset and offset times measured by the GENEActiv device. This enabled us to quantify daily activity-space exposures during the wake period preceding each night’s sleep period. For days where sleep onset occurred after 00:00, we truncated the wake exposure window to 23:59 [30].

### Linkage of neighborhood environmental data

We spatially linked all GPS points occurring during wake periods for each participant to measures representing domains of crime and physical disorder, as well as relevant neighborhood-level covariates. Table 1 includes detailed information on the data source, spatial definition, and parameterization of each exposure variable. We linked neighborhood measures to GPS points as well as the geocoded home and school addresses of each participant. Data on crime incidents for 2022-2024 were downloaded from the city of Philadelphia police department via the OpenDataPhilly portal [47]. For each GPS point, we calculated the total number of part 1 crimes occurring in the corresponding calendar year within a 660-foot buffer of the point (approximately the length of one city block). Part 1 crimes are a classification used by the FBI’s Uniform Crime Reporting program for serious, high-volume crimes that are likely to be reported to the police, including homicide, rape, aggravated assault, burglary, larceny, motor vehicle theft, and arson. There were between 82 000 and 94 000 total part 1 crime incidents in Philadelphia annually between 2022 and 2024 [47].

**Table 1:** Parameterization of neighborhood exposure measures^†^^,^^‡^

Domain represented	Data source (years)	Variable definition	Exposure definition – median (interquartile range)		
			Home buffer	School buffer	Daily activity space
Crime/safety	Philadelphia Police Department Crime Data (2022-2024)	Annual count of part 1 police-recorded crimes (serious crimes including homicide, rape, robbery, aggravated assault, burglary, larceny, motor vehicle theft, arson)	64.5 (62.0)	56.5 (84.0)	73.7 (65.9)
Physical disorder	City of Philadelphia administrative datasets (2024)	Number of vacant lots	5.0 (9.0)	1.0 (6.0)	3.7 (7.1)

^†^Neighborhood crime and vacant lot exposures were linked to each GPS location point by taking the count within a 660-foot buffer (approximately the length of a city block) of the point. This was used to create three neighborhood definitions used in this analysis. Home and school neighborhood measures were **person-level exposures** (e.g. one value per participant) that reflect the value within a 660-foot buffer of the geocoded home/school address. Daily activity space measures were calculated by taking the average across all GPS points between the participants’ actigraphy-assessed wake and sleep onset times for each day. They reflect time-weighted average exposures to crime and vacant lots at the **person-day level** (up to 14 per participant).

^‡^Interpretation for neighborhood crime exposure measures: the median adolescent had a total of 64.5 part 1 crime incidents occurring within a 660-foot radius of their home address during the calendar year of data collection and a total of 56.5 incidents within a 660-foot radius of their school address. The median number of crime incidents in adolescents’ daily activity spaces, which average across all GPS points and give more weight to area in which adolescents spend more time, was 73.7 incidents.

Physical disorder was quantified using data on vacant lot locations in the City of Philadelphia extracted from the OpenDataPhilly portal, which included the location of 28 838 properties across Philadelphia likely to be a vacant lot based on an assessment of City of Philadelphia administrative datasets [48]. We calculated the number of vacant lots within a 660-foot buffer of each GPS point. We selected vacant lots as an indicator of neighborhood physical disorder in consideration of both data availability and conceptual importance. While neighborhood physical disorder is often measured using self-report, systematic social observations, or Google Street View imagery to rate neighborhoods for the presence of multiple features (e.g. vacant lots, trash, graffiti, broken windows [49]), this approach was not feasible for our study, as it would require ratings for the full activity paths of each adolescent over the 14-day study period. Prior studies have described associations between vacant, unmaintained land and poorer physical and mental health [50–52], and remediation of vacant lots has been found to improve mental health outcomes among nearby residents [53], supporting the potential for vacant lots to impact sleep outcomes through increasing perceived stress and vigilance.

As neighborhood confounders, we included five activity-space measures: economic deprivation, social fragmentation, tree canopy cover, walkability, and recreational facilities (Table S1). These potential confounders were selected as they have been shown to be associated with adolescent sleep outcomes in prior studies [5, 22, 54, 55] and/or reflect aspects of the neighborhood physical or social environment that may influence crime and physical disorder [24, 56]. Economic deprivation and social fragmentation indices were constructed using data from the American Community Survey following similar procedures used in prior investigations of adolescent sleep [22, 54]. The percent urban tree canopy cover was extracted from the National Agriculture Imagery Program Urban Tree Canopy Assessment [57], while walkability was assessed using the U.S. Environmental Protection Agency’s National Walkability Index [58] and the count of recreational facilities (e.g. fitness centers, pools, sports clubs) was extracted from the National Neighborhood Data Archive Recreational Facilities dataset [59]. These measures were spatially linked to each GPS point based on the census tract that contained the point, and to the census tract containing each participants’ home and school.

After assigning neighborhood exposure and confounder values to each GPS point, we calculated person-day-level values for each environmental measure by averaging the values of all points within each participant’s wake period on each day of follow-up (up to 14 person-days per participant). This process generated daily activity-space exposure measures for each participant that was temporally weighted, such that areas in which participants spent a greater amount of time contributed a greater number of points to the daily average. The distribution of these person-day-level activity-space measures is presented in Table 1 for crime and vacant lots and Table S1 for the neighborhood confounders.

### Covariates

Covariates included a priori determined potential confounders of the relationship between activity-space crime and physical disorder and adolescent sleep patterns, including age, sex at birth, race/ethnicity, family socioeconomic status, travel pattern and neighborhood environmental variables, school night (versus weekend night) status, and month (to account for seasonality). On the baseline survey, adolescents self-reported their sex at birth (female, male) and their race and ethnicity according to standard National Institutes of Health (NIH) categories. We categorized race/ethnicity as follows for the analysis: non-Hispanic Black, non-Hispanic white, Hispanic, non-Hispanic other race (combined due to the small number of participants reporting other race categories). Race/ethnicity was included as markers for potential exposure to structural racism. Parent-reported household income, parents’ highest educational attainment and marital status were included as measures of family socioeconomic status. Household income was classified as: less than $25 000, $25 000-49 999, $50 000-99 999, and $100 000 or more. Parental education was classified as: high school or less, some college/associate’s degree, bachelor’s degree, master/professional degree. Marital status was categorized as: married/living with a partner versus not. In addition, adolescents’ daily commute to school and amount of time spent away from home might impact both sleep and daily neighborhood exposures, so distance from home to school (in meters) and percentage of GPS points that were > 250 meters from home were included as covariates. Also, as described above, daily activity-space neighborhood economic deprivation, social fragmentation, tree canopy cover, walkability, and recreational facilities were included as environmental confounders.

### Statistical analysis

In total, 195 adolescents completed the baseline survey and were asked to download the Aware location-tracking app. Among them, 8 participants did not download the app, 8 were excluded due to insufficient GPS data (i.e. not having a least one day with >10 hours of GPS data collected), and one was excluded due to travelling out of the study region for the majority of the data collection period, leaving 178 participants with GPS data. The analytic sample for the current analysis included participants who had at least one day of overlapping GPS and actigraphy data and complete activity-space exposure and covariate data, for a total of 142 participants with 1347 sleep periods (Figure 1). Included participants were demographically similar to the overall Teen NAPS study population (Table S2).

**Figure 1:**
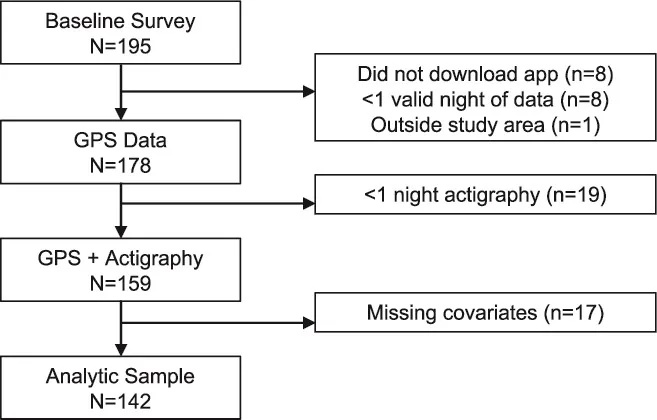
Study inclusion.

The distribution of sleep outcomes, neighborhood environmental exposures, and covariates was examined across participants. We calculated Spearman correlation coefficients between the daily activity-space measures for descriptive purposes (Table S3). We also calculated Spearman correlation coefficients between activity space crime and vacant lot exposures and the sleep outcomes of interest at the person-level (averaged across nights, Table S4) to examine bivariate correlations. To estimate associations of home, school, and daily activity-space exposure to crime and vacant lots with adolescent sleep duration, timing, and efficiency, we used multivariable linear mixed effects models. Models included participant random intercepts to account for clustering of days within adolescents and an autoregressive correlation structure to account for correlations between adjacent days of follow-up. To estimate associations with the dichotomous outcomes of insufficient sleep and very short sleep, we used modified Poisson regression with robust variance estimates [60]. In all models, neighborhood variables were scaled to reflect associations for an interquartile range (IQR) difference in exposure due to the skewed nature of the data (Figure S1). Mixed effects models were adjusted for both person-level and day-level covariates. Person-level covariates included adolescent age, sex, race/ethnicity, parent-reported household income, parental education, parental marital status, distances between home and school, and percent of GPS points that were > 250 m from home. Day-level covariates included school night status, month (considered a day-level variable because 34% of adolescents had data collection periods that crossed months), and activity-space economic deprivation, social fragmentation, tree canopy cover, walkability, and recreational facilities. Daily activity-space crime and vacant lot exposures were moderately correlated (Spearman correlation coefficient: 0.589, Table S3) and were included in mutually adjusted models.

To estimate within-person associations of day-to-day changes in activity-space exposure to neighborhood crime and vacant lots with sleep outcomes, we used models that included a fixed effect for each adolescent. Including individual fixed effects enabled us to examine whether adolescents’ sleep patterns differed on days when their activity space exposure to crime and vacant lots exceeded their own average level. By exclusively using within-person variation, fixed-effects models treat each individual as their own control and inherently control for confounding variables that do not change over time, both measured and unmeasured [16]. Models were adjusted for the time-varying day-level covariates described above.

As a sensitivity analysis, we repeated the models described above among the subset of participants who had at least 5 days of simultaneous GPS and actigraphy data (*n* = 121 adolescents), as inclusion of at least 5 days may improve reliability of actigraphy-based sleep estimates [61]. Analyses were completed in R version 4.4.0 (Vienna, Austria) and Stata version 18.0 (StataCorp, College Station, TX).

## Results

### Description of the study population

Participants had an average of 9.5 days of simultaneous GPS tracking and actigraphy sleep data (SD: 4.0). Table 2 summarizes participant characteristics and sleep outcomes. The mean age was 16.2 years (SD: 0.9) and 59.2% were female. 50.0% reported their race/ethnicity as non-Hispanic Black, 28.9% as non-Hispanic white, 12.7% as Hispanic, and 8.5% as another race/ethnicity. About 14.1% had household incomes of <$25 000 while 40.8% reported household income ≥$100 000. 30.3% of adolescents had parents with a graduate or professional degree. Mean sleep duration was 390.8 minutes (6.5 hours; SD: 109.4), with insufficient sleep (<8 hours) on 81.8% of nights and very short sleep (<6 hours) on 40.5% of nights.

**Table 2:** Study population characteristics (*N* = 142)

	*N* (%)
Age – mean (SD)	16.2 (0.9)
Sex	
Female	84 (59.2)
Male	58 (40.8)
Self-reported race/ethnicity	
Black	71 (50.0)
Hispanic	18 (12.7)
White	41 (28.9)
Other race/ethnicity^†^	12 (8.5)
Household income	
< $25 000	20 (14.1)
$25 000-$49 999	24 (16.9)
$50 000-$99 999	40 (28.2)
≥ $100 000	58 (40.8)
Parental education	
≤ High school	24 (16.9)
Some college/associate’s	48 (33.8)
Bachelor’s degree	27 (19.0)
Graduate degree	43 (30.3)
Parent marital status	
Married/living with partner	69 (48.6)
Not married/living with partner	73 (51.4)
Distance between home and school (km) – Mean (SD)	5.3 (4.3)
Percent of time spent >250 m from home – Mean (SD)	67.0 (17.8)
**Sleep outcomes (night-level)** ^‡^	
Duration (minutes) – Mean (SD)	390.8 (109.4)
Onset (minutes from 00:00) – Mean (SD)	13.1 (122.0)
Offset (minutes from 00:00) – Mean (SD)	488.3 (134.1)
Sleep efficiency (percentage)	83.0 (8.6)
Insufficient Sleep (<8 hours)	1102 (81.8)
Very short sleep (<6 hours)	545 (40.5)

† Includes Asian (*n* = 3) and adolescents reporting multiple race/ethnicities (*n* = 9)

^‡^Sleep outcomes assessed at the night level; 1347 nights from 142 adolescents were included.

On average, 44.4% of GPS points recorded during adolescents’ wake periods were at locations >250 meters from home and 46.9% were located outside adolescents’ home census tract. The median annual count of serious crimes in adolescents’ daily activity space was 73.7 (IQR: 65.9), while the median number of vacant lots was 3.7 (IQR: 7.1). Daily activity-space exposure to serious crimes (e.g. homicide, robbery, assault) was higher on average than exposure levels at adolescents’ home and school locations, while exposure to vacant lots in adolescents’ activity spaces was lower than home but higher than school (Table 1). The distribution of daily activity-space crime and vacant lot exposures varied across adolescents of differing sociodemographic characteristics (Table S5). Adolescents who reported their race/ethnicity as Non-Hispanic White and those with household incomes >$100 000, more highly-educated parents, and married/co-habituating parents had lower exposure to both neighborhood crime and vacant lots than those who reported other racial/ethnic identities and lower income/education/single-parent homes.

### Associations of crime with sleep outcomes

Table 3 presents the associations of home, school, and daily activity-space crime and vacant lot exposures with adolescent sleep patterns using the two modeling strategies described above. An IQR increase in crime exposure around adolescents’ home address was associated with 16-minute shorter sleep duration (95% CI: −27.7, −4.3) and 21-minute earlier sleep offset timing (95% CI: −38.4, −3.5), as well as 4% higher risk of insufficient sleep (RR: 1.04, 95% CI: 1.01, 1.07) and 17% higher risk of very short sleep (RR: 1.17, 95% CI: 1.07, 1.29), but not with differences in sleep onset timing or sleep efficiency. An IQR increase in crime exposure around adolescents’ schools was associated with similarly shorter sleep duration and higher risk of short and very short sleep (Table 3), as well as 14-minute later sleep onset timing (95% CI: 1.0 26.9), but not with offset timing or efficiency. When considering time-weighted crime exposures aggregated in adolescents’ daily activity spaces, an IQR increase in crime exposure was associated with 13.6-minute earlier sleep offset timing (95% CI: −25.5, −1.7), 4% higher risk of insufficient sleep (95% CI: 1.00, 1.08) and 10% higher risk of very short sleep (RR: 1.10, 95% CI: 0.99, 1.22), although the latter confidence interval crossed the null. Activity space exposure was not associated with sleep duration, onset timing, or efficiency. In models examining within-person associations, adolescent sleep outcomes did not differ on days where their activity space exposure to crime was higher than their own average level (Table 3).

**Table 3:** Associations of neighborhood crime and vacant lots with adolescent sleep patterns^†^^,^^‡^^,^^§^^,^^||^

	Duration (minutes) – β (95% CI)	Onset (minutes) – β (95% CI)	Offset (minutes) – β (95% CI)	Efficiency (%) – β (95% CI)	Insufficient sleep (>8 hours) – RR (95% CI)	Very short sleep (<6 hours) – RR (95% CI)
Crime						
In Home Buffer	−16.0 (−27.7, −4.3)^*^	3.9 (−15.7, 23.5)	-21.0 (−38.4, −3.5)^*^	0.5 (−0.7, 1.7)	1.04 (1.01, 1.07)^*^	1.17 (1.07, 1.29)^*^
In school buffer	−15.5 (−23.3, −7.6)^*^	14.0 (1.0, 26.9)^*^	−4.9 (−16.9, 7.1)	−0.3 (−1.1, 0.6)	1.04 (1.02, 1.06)^*^	1.11 (1.04, 1.18)^*^
In daily activity space	−6.7 (−16.7, 3.3)	−6.0 (−17.4, 5.4)	−13.6 (−25.5, −1.7)^*^	0.4 (−0.5, 1.2)	1.04 (1.00, 10.8)^*^	1.10 (0.99, 1.22)
Within-person	4.5 (−8.1, 17.1)	−6.1 (−19.0, 6.9)	-4.6 (-18.3, 9.2)	0.3 (−0.7, 1.3)	1.01 (0.95, 1.08)	0.95 (0.81, 1.10)
Vacant lots						
In home buffer	2.3 (−7.2, 11.8)	−2.7 (−18.5, 13.1)	4.5 (−9.6, 18.7)	0.0 (−1.0, 0.9)	1.01 (0.98, 1.04)	0.96 (0.87, 1.06)
In school buffer	−6.9 (−15.5, 1.8)	6.3 (−7.5, 20.1)	−2.9 (−15.8, 10.1)	0.2 (−0.7, 1.1)	1.02 (0.99, 1.05)	0.99 (0.90, 1.08)
In daily activity space	−16.2 (−24.9, −7.6)^*^	12.4 (1.4, 23.4)^*^	−8.0 (−19.1, 3.1)	−0.6 (−1.4, 0.1)	1.04 (1.02, 1.07)^*^	1.04 (0.94, 1.16)
Within-person	−34.2 (−49.6, −18.7)^*^	18.5 (−0.2, 37.1)	−20.1 (−40.1, −0.1)^*^	−0.7 (−1.8, 0.4)	1.10 (1.02, 1.18)^*^	1.28 (1.11, 1.48)^*^

† Results are from 4 sets of models. Model 1 examined associations for crime and vacant lot exposure within a 660 ft buffer (approximately 1 city block) around participants’ home address (person-level exposure). Model 2 examined associations for crime and vacant lot exposure within a 660 ft buffer around participants’ school address (person-level exposure). Model 3 examined associations for daily activity-space crime and vacant lot exposures (person-day-level exposure). Model 4 examined associations of within-person differences in daily activity space crime and vacant lot exposures (e.g. differences in sleep on days when adolescents had higher exposure than their own average level).

‡ Models were adjusted for potential confounders including adolescent age, sex, race/ethnicity, household income, parental education, parental marital status, percent of GPS location points located at least 250 meters from home, distance between home and school, school night status, month, and daily activity-space exposure to economic deprivation, social fragmentation, tree canopy cover, walkability, and recreational facilities. Models included participant random intercepts to account for clustering of days within adolescents and used an autoregressive correlation structure. Linear mixed models were used for sleep duration, onset, offset, and efficiency, while modified Poisson regression was used for insufficient and very short sleep.

§ Within-person associations were estimated using fixed-effects regression to estimate associations of within-person differences in activity space exposures and within-person differences in sleep from night to night. Models were adjusted for time-varying covariates (school night status, month, daily activity-space economic deprivation, social fragmentation, tree canopy cover, walkability, and recreational facilities). Linear models were used for sleep duration, onset, offset, and efficiency, while modified Poisson regression was used for insufficient and very short sleep.

|| All crime and vacant lot variables were scaled to reflect the association of an interquartile range difference.

^*^indicates *p* < 0.05.

### Associations of vacant lots with sleep outcomes

For vacant lot exposure, higher exposure levels around adolescents’ homes and schools were not associated with differences in sleep outcomes. However, an IQR increase in vacant lot exposure in adolescents’ daily activity space was associated with 16.2-minute shorter sleep duration (95% CI: −24.9, −7.6), 12.4-minute later sleep onset timing (95% CI: 1.4, 23.4), and 4% higher risk of insufficient sleep (RR: 1.04, 95% CI: 1.02, 1.07), but not with other outcomes. In the fixed-effects models, which estimated within-person associations, exposure to vacant lots that was higher than adolescents’ own average was associated with 34.2-minute shorter sleep duration (95% CI: −49.6, −18.7), 20.1-minute earlier offset (95% CI: −40.1, −0.1), 10% higher risk of insufficient sleep (RR: 1.10, 95% CI: 1.02, 1.18) and 28% higher risk of very short sleep (RR: 1.28, 95% CI: 1.11, 1.48) per IQR difference.

Tables S6–S7 present the full set of regression coefficients for the activity space and fixed-effects models. In sensitivity analyses restricted to participants with at least 5 nights of GPS and actigraphy data, results were similar (Table S8).

## Discussion

To our knowledge, this is the first study to assess associations of neighborhood crime and vacant lots across multiple settings, including home, school, and daily activity space, with adolescent sleep patterns. In this study of 15–18-year-old adolescents in Philadelphia, higher counts of serious crimes in the areas surrounding adolescents’ homes and schools were associated with shorter sleep duration and higher risk of inadequate sleep, with variable associations with sleep timing. Higher crime in adolescents’ daily activity space was associated with earlier offset timing and with higher risk of insufficient sleep. Higher activity space vacant lot exposure, but not exposure around the home or school, was associated with short sleep duration, later onset timing, and higher risk of insufficient sleep. When we examined within-person associations to determine whether daily exposures exceeding adolescents’ typical levels were associated with differences in their sleep outcomes across nights, we found higher than usual vacant lot exposure was associated with shorter sleep duration, earlier offset and higher risk of insufficient and very short sleep. Notably, a majority of adolescents in the study had insufficient sleep as measured by actigraphy, consistent with national data indicating suboptimal sleep duration among U.S. high school students [2].

Neighborhood crime and physical disorder are hypothesized to impact sleep through increasing levels of vigilance and anxiety, which may make it harder to fall asleep or stay asleep [9]. Prior studies in adolescent populations have found that lower perceived safety and greater community violence concerns are associated with more adverse sleep patterns and self-reported sleep problems [4, 9–13], although few studies have examined objective indicators of crime or physical disorder. Two studies have used actigraphy to examine within-person associations of daily fluctuations in crime in adolescents’ home neighborhoods with subsequent sleep outcomes [14, 15]. In a study among adolescents in a large Midwestern city, adolescents had later bedtimes on nights following a violent crime in their neighborhood [14]. A second study using a similar design among adolescents in New York City found that sleep efficiency decreased by nearly 1% on days where ≥2 violent crimes had happened in an adolescents’ neighborhood, while adolescents living in neighborhoods that had higher crime levels on average had later bedtimes and earlier wake times [15].

To our knowledge, the associations of objectively-measured crime in the area surrounding adolescents’ schools, or in their daily activity space, with sleep outcomes have not been previously assessed. A school-based survey of adolescents in Florida assessed adolescents’ perceptions of safety at school and in their home neighborhoods and found that adolescents who perceived their school as less safe were more likely to have self-reported insufficient sleep, although the association was much stronger when adolescents also felt unsafe in their home neighborhoods [4]. In our study, crime levels near home, school, and in adolescents’ activity spaces were associated with various sleep outcomes, but within-person models did not provide evidence for poorer sleep outcomes on nights where adolescents spent more time in higher crime areas. A possible explanation is that crime levels might have more of a long-term effect on sleep rather than an acute impact. Alternatively, it is possible that the associations between home, school, and activity space crime levels and sleep outcomes, such as earlier sleep offset timing and risk of insufficient sleep, are confounded by other unmeasured individual or environmental factors such as noise pollution or household crowding [15].

The limited prior research on neighborhood physical disorder and adolescent sleep has been mixed [6, 22, 23], and prior studies have not examined vacant lots specifically. Vacant lots, which may be filled with trash and overgrown vegetation and are often concentrated in neighborhoods impacted by redlining and economic disinvestment, are persistent stressors that may increase feelings of fear and reduce social cohesion among neighborhood residents [50], and have been shown to be associated with poorer mental health [51]. A randomized controlled trial of vacant lot remediation in Philadelphia found that converting vacant lots into cleaned, green spaces improved depressive symptoms among nearby residents [53]. Our findings that adolescents had shorter sleep, earlier offset timing, and higher risk of insufficient and very short sleep on days when they spent more time in areas with higher levels of vacant lots suggests a potential impact of vacant lots on sleep, although the observational study design limits causal inferences, and future experimental work is needed to determine the impact of remediating vacant lots on adolescent sleep outcomes.

Notably, most prior studies of crime, physical disorder, and adolescent sleep have examined exposures in their residential neighborhood alone [3]. In our sample, nearly 50% of adolescents’ waking observations occurred outside the home census tract (a commonly-used definition of an adolescent’s “neighborhood”), suggesting substantial misclassification may occur when non-home locations are ignored. Adolescents may spend much of their day outside the home neighborhood for school, work, or other daily activities. Our findings that associations of crime and vacant lots with sleep outcomes differed between home, school, and activity space contexts underscores the potential importance of considering exposures throughout daily activities, beyond the residential neighborhood. For example, our finding that adolescents had more adverse sleep patterns on days when their exposure to vacant lots was higher than usual suggests a potential impact of locations outside the home or school context, for example areas where adolescents are passing through or spending time on particular days. Two prior studies in adult populations have similarly used GPS tracking to derive activity-space exposures and examined associations with sleep outcomes. A study among sexual minority men in New York City found weekly activity-space-based crime was not associated with self-reported sleep outcomes [29], while a study among adult nurses across the United States found daily smartphone GPS-based measures of walkability were not associated with actigraphy-derived sleep duration or efficiency [30]. While many prior studies have used research grade location-tracking devices, smartphone GPS tracking, as used in our study, is emerging as a promising strategy to improve acceptability and sustainability, overcoming limitations like the requirement of charging and wearing a separate device. A recent study comparing smartphone GPS tracking to research grade GPS devices found that when GPS-based measures are aggregated to the day-level, as in our study, activity-space measures derived from smartphone and research-grade GPS devices were similar [62].

Strengths of this study include intensive daily assessment of both sleep outcomes and activity-space environmental exposures, an approach just beginning to be applied to sleep research. Limitations include the observational, cross-sectional design, which limits our ability to make causal interpretations. In addition, our study population of healthy adolescents from a single U.S city may not generalize to other areas, and our study population had higher household income levels than adolescents in the city of Philadelphia overall. Although use of an activity-space framework improves exposure assessment by accounting for mobility, there may be measurement error as some environmental variables were linked to GPS points at the census tract level, and such administrative boundaries may not match participants’ conceptions of neighborhood boundaries. In addition, although we included a robust set of neighborhood features informed by prior literature, we lacked information on other activity-space features (e.g. noise, social cohesion) that might influence sleep, and there may be residual confounding by other unmeasured individual or environmental factors. Finally, the crime variable we used was aggregated by calendar year, so we did not examine day-to-day fluctuations in crime within neighborhoods as some prior studies have done [14, 15].

## Conclusion

Associations of neighborhood crime and vacant lots with adolescents’ sleep outcomes varied across home, school, and daily activity spaces, with adolescents having particularly adverse sleep outcomes on nights where their exposure to vacant lots was higher than their usual level. Smartphone GPS tracking facilitates assessment of more nuanced measures of neighborhood environmental exposures that account for mobility and enable examination of the relation of activity-space exposures with adolescent sleep health.

## Supplementary Material

Supplemental_Appendix_zsag226

## Data Availability

The data underlying this article cannot be shared publicly due to the sensitive nature of the location tracking data and to protect privacy of the individuals who participated in the study. A deidentified version will be shared on reasonable request to the corresponding author.
