# Supplementary material for: Associations between daily neighborhood crime and physical disorder exposure and sleep patterns in adolescents
Source: Sleep. 2026 Aug 19;49(11):zsag226. doi: 10.1093/sleep/zsag226 (PMC13747340; doi:10.1093/sleep/zsag226)
Supplement: Supplementary file 1 [file supplemental_appendix_zsag226.docx]

**Supplemental Appendix**

**Associations Between Daily Neighborhood Crime and Physical Disorder Exposure and Sleep Patterns in Adolescents**

Stephanie L. Mayne,^1,2,3^ Gabrielle DiFiore,^1^ Alexander G. Fiks,^1,2,3^ Hannah Martin,^1^ Vicky Tam,^4^ Jonathan A. Mitchell,^2,5^ Ian Barnett,^6^ Douglas J. Wiebe,^7^ Mathias Basner,^8^ Karen Glanz^3,6,9^

**Affiliations**:

^1^Clinical Futures, PolicyLab, and the Possibilities Project, Children’s Hospital of Philadelphia, Philadelphia, Pennsylvania, United States

^2^Department of Pediatrics, University of Pennsylvania Perelman School of Medicine, Philadelphia, Pennsylvania, United States

^3^Leonard Davis Institute for Healthcare Economics, University of Pennsylvania Perelman School of Medicine, Philadelphia, Pennsylvania, United States

^4^Data Science and Biostatistics Unit, Children’s Hospital of Philadelphia, Philadelphia, Pennsylvania, United States

^5^Division of Gastroenterology, Hepatology, and Nutrition, Children’s Hospital of Philadelphia, Philadelphia, Pennsylvania, United States

^6^Department of Biostatistics, Epidemiology, and Informatics, University of Pennsylvania Perelman School of Medicine, Philadelphia, Pennsylvania, United States

^7^Department of Epidemiology, University of Michigan School of Public Health, Ann Arbor, Michigan, United States

^8^Department of Psychiatry, University of Pennsylvania Perelman School of Medicine, Philadelphia, Pennsylvania, United States

^9^School of Nursing, University of Pennsylvania, Philadelphia, Pennsylvania, United States

**Corresponding Author**: Stephanie Mayne, PhD, MHS, Children’s Hospital of Philadelphia (CHOP), 2716 South Street, 10-471, Philadelphia PA 19146, [maynes@chop.edu](mailto:maynes@chop.edu), 267-426-0192

**Supplemental Methods: Cleaning and Processing of Actigraphy Data**

Raw accelerometry data from returned GENEActiv devices were downloaded as .bin files and processed using GGIR version 3.1.2 in R version 4.4.0 with the following settings:

GGIR Parts 4 and 5 were examined to flag nights of actigraphy data with suspected poor data quality. Data quality criteria were determined through consultation with actigraphy experts and review of the available data to generate a set of criteria likely to flag nights with poor data quality. Similar filters were applied in a recent validation study (Chen et al, 2025). We flagged data meeting the following criteria (GGIR variable names italicized):

Percent non-wear (*nonwear_perc_spt_p5*, Part 5) >30%

Fraction of the night invalid (*fraction_night_invalid*, Part 4) >30%

Sleep duration (*SleepDurationInSpt*, Part 4) <2 hours

Sleep offset time (*wakeup_p4*, Part 4) <01:00AM

Sleep efficiency (*SleepDurationInSpt / SptDuration*, Part 4) = 100%

The actigrams for nights flagged by these criteria were manually inspected by a study team member (SM or GD), with review by a second reviewer when needed for a consensus decision. In total, 173 nights were flagged and reviewed, with a total of 133 nights determined to be of insufficient quality and excluded. We subsequently excluded 1 additional where the primary sleep period appeared to be valid but occurred during the daytime (i.e., sleep offset occurring before midnight), as our study question focused on the relationship of daily exposures with nightly sleep.

**Reference:**

Chen PW, Jansen EC, Cielo CM, et al. Performance of an Automated Sleep Scoring Approach for Actigraphy Data in Children and Adolescents. Sleep. Sep 19 2025;doi:10.1093/sleep/zsaf282

**Supplemental Methods: Cleaning and Processing of GPS Data**

***Data Cleaning:***

Latitude and longitude coordinates were collected at approximately 60-second intervals throughout the 14-day data collection period using the study-specific Aware app. In addition to timestamped location coordinates, the app captured estimated accuracy (in meters), bearing, speed, and altitude. Data were securely transferred from adolescents’ smartphones to the study’s Amazon Web Services (AWS) server located at the University of Pennsylvania. Upon downloading the dataset for analysis, the following data cleaning steps were taken:

1) Excluding points collected before or after each participant’s 14-day data collection window
2) Excluding points with inaccurate or implausible values:
   a. Latitude/longitude coded as 0 (0.1% of coordinates)
   b. Accuracy >100 meters (3.5% of coordinates)
   c. Speed > 130 kilometers/hour (<0.1% of coordinates)
   d. Elevation change > 1000 meters (<0.1% of coordinates)
3) Deduplicating points to the minute-level (for some adolescents, multiple coordinates were captured simultaneously, so we prioritized the coordinate pair with the highest accuracy at each time point)
4) Excluding person-days with less than 10 hours (600 minutes) of valid GPS data (n=373 person-days from 7 participants)
5) Excluded a participant who travelled out of state for most of the study period (n=10 person-days from 1 participant)

Participants had GPS location data available for a total of 2,197,352 out of 2,852,720 minutes of participation in the study, with a median of 17,311 minutes of coverage (85.9%). In a sensitivity analysis, we imputed missing GPS points if the gap between successive points was between 2 and 60 minutes and the successive coordinates were <100 meters apart. If these criteria were met (1.3% of observed points), we carried forward the last known location to fill in the missing locations. This added an additional 63,175 location points to the analysis. After imputation, results were similar to the main analysis, so the unimputed results are presented.

***Linkage of Neighborhood Exposure Data:***

After cleaning the GPS data, we used ArcGIS Pro to link neighborhood exposure measures to each GPS point based on the spatial aggregations described in Table 1 (660 ft buffer around the point - i.e. one city block - for crime and vacant lots; census tract for economic deprivation, social fragmentation, tree canopy cover, walkability, and recreational facilities).

***Calculation of Wake Period Activity-Space Exposure Measures***

We then merged the GPS data with adolescents’ GENEActiv actigraphy data based on calendar date. We used actigraphy-derived sleep onset and offset times to identify GPS points occurring during the wake period for each person-day in the analytic dataset. For example, if the wake window for January 01 was 06:30 A.M. to 23:30 P.M., all GPS points occurring between those times were coded as wake. For days where sleep onset occurred after midnight (00:00 A.M.), the wake exposure window was truncated at 23:59 P.M. We then averaged the values of each neighborhood measure for GPS points within the wake period on each person day to create daily activity-space measures of exposure to crime, vacant lots, economic deprivation, social fragmentation, tree canopy cover, walkability, and recreational facilities. This was done separately for each person-day, such that adolescents could have up to 14 different values for each measure.

Thus, daily wake period activity-space exposures could be examined in relation to nightly sleep outcomes, e.g., the average exposure to vacant lots in adolescents’ activity space during the wake period of January 01 would be linked with the main sleep period occurring the night of January 01 to January 02.

**Supplemental Table 1. Parameterization of Neighborhood Confounders**

| **Domain Represented** | **Data source (Years)** | **Variable Definition** | **Exposure Definition –**  **Median (Interquartile Range)** | | |
| --- | --- | --- | --- | --- | --- |
|  |  |  | **Home**  **Census Tract** | **School Census**  **Tract** | **Daily Activity Space** |
| Economic Disadvantage | American Community Survey (2019-2023 5-year estimates) | Index of three z-score transformed census variables: percent poverty, percent receiving public assistance, and median household income (reverse coded)^1^ | 0.1 (3.3) | 0.1 (4.3) | -0.1 (3.6) |
| Social Fragmentation | American Community Survey (2019-2023 5-year estimates) | Index of three z-score transformed census variables: percent vacant households, percent owner occupied household (reverse coded), percent residents who moved within past year^1^ | -0.6 (2.5) | 0.2 (3.1) | -0.3 (2.9) |
| Green Space | National Agriculture Imagery Program Tree Canopy Assessment (2022-2023) | Percent tree canopy cover | 15.0 (13.5) | 12.8 (22.6) | 16.7 (13.8) |
| Walkability | Environmental Protection Agency National Walkability Index (2019) | Index ranking U.S. census tracts according to intersection density, proximity to transit stops, and diversity of land uses (e.g., employment mix, employment and household mix) with possible range 1-20 | 15.0 (2.3) | 15.5 (3.0) | 15.1 (2.0) |
| Recreational Facilities | National Neighborhood Data Archive Recreational Establishments dataset (2022) | Count of recreational services (e.g., fitness centers, golf courses, skating rinks, pools, membership sports clubs, specialized recreational establishments | 1.0 (2.0) | 2.0 (4.0) | 2.0 (2.6) |

^1^Neighborhood confounder measures were linked to each GPS location point based on the census tract it was located in.

^2^For economic deprivation and social fragmentation index scores, each component was standardized to a z-score (mean: 0.0, SD: 1.0) and then summed, i.e. a census tract with the mean level for all three components would have a score of 0. A higher score indicates a greater level of economic deprivation or social fragmentation, respectively.

**Supplemental Table 2. Demographics of Analytic Sample in Comparison to All Teen NAPS Participants and Those with Valid GPS Data^1,2^**

| **Sociodemographics** | **Analytic Sample**  **N (%)** | **Baseline Survey Sample** | **GPS Sample** |
| --- | --- | --- | --- |
| N | 142 | 195 | 178 |
| Age – Mean (SD) | 16.2 (0.9) | 16.4 (1.0) | 16.4 (1.0) |
| Sex |  |  |  |
| Female | 84 (59.2) | 115 (59.0) | 104 (58.4) |
| Male | 58 (40.8) | 80 (41.0) | 74 (41.6) |
| Self-reported Race/Ethnicity |  |  |  |
| Non-Hispanic Black | 71 (50.0) | 98 (50.3) | 88 (49.4) |
| Hispanic | 18 (12.7) | 24 (12.3) | 22 (12.4) |
| Non-Hispanic White | 41 (28.9) | 55 (28.2) | 51 (28.6) |
| Other race/ethnicity | 12 (8.5) | 18 (9.2) | 17 (9.6) |
| Household Income |  |  |  |
| <$25,000 | 20 (14.1) | 24 (14.0) | 23 (14.6) |
| $25,000-$49,999 | 24 (16.9) | 32 (18.7) | 29 (18.3) |
| $50,000-$99,999 | 40 (28.2) | 48 (28.1) | 42 (26.6) |
| ≥$100,000 | 58 (40.8) | 67 (39.2) | 64 (40.5) |
| Parental Education |  |  |  |
| ≤High School | 24 (16.9) | 31 (18.1) | 29 (18.3) |
| Some College/Associate’s | 48 (33.8) | 55 (32.2) | 52 (32.9) |
| Bachelor’s Degree | 27 (19.0) | 35 (20.5) | 30 (19.0) |
| Graduate Degree | 43 (30.3) | 50 (29.2) | 47 (29.8) |

^1^Baseline survey sample included 195 adolescents who completed the baseline survey upon enrollment into the study. GPS sample included 178 adolescents who successfully downloaded the GPS tracking app and had at least 1 day of valid GPS data collection (and excluded 1 adolescent who was outside the study region for the majority of the study period). The analytic sample included 142 adolescents with at least 1 day of overlapping, valid GPS and actigraphy data and complete covariate and exposure data.

^2^Household income and parental education were only available for adolescents whose parents completed the baseline survey (n=171 out of the 195 who completed the baseline survey and n=158 out of the 178 with valid GPS data)

**Supplemental Figure 1. Distribution of Daily Activity Space Crime and Vacant Lot Exposures.** Variables are at the person-day level (up to 14 observations per participant) and reflect the average level of crime incidents or vacant lots within a 660ft buffer of all GPS points within adolescents’ actigraphy-measured wake and sleep times on each day.

**Supplemental Table 3. Spearman Correlations Between Daily Activity-Space Neighborhood Measures**

|  | **Crime** | **Vacant Lots** | **Econ Dep** | **Social Frag** | **Tree Canopy** | **Walk-ability** | **Rec Fac** |
| --- | --- | --- | --- | --- | --- | --- | --- |
| **Crime** | 1.0 |  |  |  |  |  |  |
| **Vacant Lots** | 0.589 | 1.0 |  |  |  |  |  |
| **Econ Dep** | 0.256 | 0.447 | 1.0 |  |  |  |  |
| **Social Frag** | 0.432 | 0.191 | 0.252 | 1.0 |  |  |  |
| **Tree Canopy** | -0.464 | -0.449 | -0.224 | -0.044 | 1.0 |  |  |
| **Walkability** | 0.316 | -0.014 | -0.151 | 0.400 | -0.347 | 1.0 |  |
| **Recreational Facilities** | 0.125 | -0.145 | -0.518 | 0.190 | -0.016 | 0.413 | 1.0 |

**Supplemental Table 4. Spearman Correlations of Daily Activity Space Neighborhood Exposures and Sleep Parameters at the Person-Level^1^**

|  | **Crime** | **Vacant** | **Dur** | **On** | **Off** | **Eff** | **<8 hrs** | **<6 hrs** |
| --- | --- | --- | --- | --- | --- | --- | --- | --- |
| **Crime** | 1.0 |  |  |  |  |  |  |  |
| **Vacant Lots** | 0.628 | 1.0 |  |  |  |  |  |  |
| **Duration** | -0.152 | -0.138 | 1.0 |  |  |  |  |  |
| **Onset** | -0.020 | 0.096 | -0.355 | 1.0 |  |  |  |  |
| **Offset** | -0.057 | 0.070 | 0.282 | 0.657 | 1.0 |  |  |  |
| **Efficiency** | -0.093 | -0.173 | 0.329 | 0.167 | 0.025 | 1.0 |  |  |
| **Insufficient Sleep (<8hrs)** | 0.098 | 0.014 | -0.771 | 0.184 | -0.288 | -0.263 | 1.0 |  |
| **Very Short Sleep (<6hrs)** | 0.176 | 0.166 | -0.848 | 0.341 | -0.164 | -0.329 | 0.561 | 1.0 |

^1^Variables were averaged across days to create person-level averages.

**Supplemental Table 5. Distribution of Daily Activity-Space Crime and Vacant Lot Exposures by Participant Characteristics**

|  | **Crime**  Median (IQR) | **Vacant Lots**  Median (IQR) |
| --- | --- | --- |
| Overall | 73.7 (65.9) | 3.7 (7.1) |
| **Demographics:** |  |  |
| Age |  |  |
| 15 | 65.4 (70.0) | 3.3 (6.1) |
| 16 | 74.6 (60.6) | 4.1 (6.9) |
| 17 | 76.6 (67.9) | 3.1 (7.7) |
| 18 | 88.1 (61.9) | 4.2 (10.3) |
| Sex |  |  |
| Female | 72.8 (67.1) | 3.4 (5.8) |
| Male | 75.1 (63.8) | 4.2 (8.5) |
| Race/ethnicity |  |  |
| Hispanic | 97.6 (75.4) | 4.4 (5.1) |
| Non-Hispanic Black | 83.6 (59.2) | 7.0 (10.2) |
| Non-Hispanic White | 51.6 (66.3) | 1.7 (3.2) |
| Non-Hispanic Other | 75.4 (52.3) | 3.0 (4.0) |
| Household Income |  |  |
| <$25,000 | 77.5 (53.1) | 5.0 (5.7) |
| $25,000-$49,000 | 93.8 (69.3) | 9.8 (9.4) |
| $50,000-$99,000 | 81.1 (61.6) | 4.0 (8.2) |
| $100,000 + | 55.3 (66.7) | 2.0 (3.8) |
| Parent Education |  |  |
| High school or less | 91.8 (57.7) | 5.3 (8.9) |
| Some college | 73.9 (53.9) | 5.1 (8.0) |
| Bachelor’s | 67.5 (77.3) | 2.1 (7.2) |
| Graduate | 62.8 (71.7) | 2.2 (3.9) |
| Parent Marital Status |  |  |
| Married/Living with Partner | 60.7 (69.8) | 2.3 (4.9) |
| Not married/living with partner | 82.3 (66.8) | 5.0 (9.2) |
| Night Type |  |  |
| School Night | 72.6 (64.6) | 3.6 (6.5) |
| Non-School Night | 76.1 (66.7) | 4.0 (8.2) |

**Supplemental Table 6. Full Regression Model Output- Daily Activity Space Models^1,2^**

|  | Duration (minutes) – β (95% CI) | Onset (minutes) – β (95% CI) | Offset (minutes) – β (95% CI) | Efficiency (%) - β (95% CI) | Insufficient Sleep (>8 hours) – RR (95% CI) | Very Short Sleep (<6 hours)  – RR (95% CI) |
| --- | --- | --- | --- | --- | --- | --- |
| Crime | -6.7  (-17.7, 3.3) | -6.0  (-17.4, 5.4) | **-13.6**  **(-25.5, -1.7)*** | 0.4  (-0.5, 1.2) | **1.04**  **(1.00, 1.08)*** | 1.10  (0.99, 1.22) |
| Vacant lots | **-16.2**  **(-24.9, -7.6)*** | **12.4**  **(1.4, 23.4)*** | -8.0  (-19.1, 3.1) | -0.6  (-1.4, 0.1) | **1.04**  **(1.02, 1.07)*** | 1.04  (0.94, 1.16) |
| Age (per year) | -4.3  (-13.6, 5.1) | -7.0  (-22.4, 8.3) | -7.6  (-21.2, 6.0) | -0.8  (-1.7, 0.2) | 1.02  (0.99, 1.04) | 0.97  (0.88, 1.08) |
| Sex (versus Female) | **-28.7**  **(-45.1, -12.3)*** | 24.9  (-2.0, 51.8) | -1.1  (-25.1, 22.8) | **-1.8**  **(-3.5, -0.1)*** | **1.13**  **(1.08, 1.19)*** | **1.42**  **(1.19, 1.71)*** |
| Race/ethnicity (versus non-Hispanic Black) |  |  |  |  |  |  |
| Hispanic | 3.9  (-22.4, 30.1) | 41.0  (-2.2, 84.2) | 16.7  (-21.7, 55.2) | **3.3**  **(0.6, 5.9)*** | 0.95  (0.88, 1.02) | 0.85  (0.66, 1.09) |
| Non-Hispanic White | 9.5  (-14.7, 33.7) | 0.7  (-38.1, 39.6) | -26.8  (-61.7, 8.1) | **4.5**  **(2.1, 6.9)*** | 0.99  (0.93, 1.07) | **0.72**  **(0.54, 0.96)*** |
| Other | -17.5  (-49.6, 14.6) | 37.7  (-13.7, 89.1) | -21.6  (-67.7, 24.6) | **4.9**  **(1.7, 8.1)*** | 1.08  (0.99, 1.19) | 1.25  (0.85, 1.85) |
| Household Income (versus <$25,000 |  |  |  |  |  |  |
| $25,000-$49,999 | 2.8  (-27.4, 33.0) | 30.4  (-18.6, 79.4) | 26.2  (-17.7, 70.1) | 1.4  (-1.7, 4.4) | 0.98  (0.89, 1.07) | 1.15  (0.86, 1.54) |
| $50,000-$99,999 | -17.1  (-48.1, 13.9) | -3.1  (-52.5, 46.3) | -18.9  (-63.5, 25.6) | -0.4  (-3.5, 2.7) | 1.00  (0.92, 1.09) | 1.16  (0.85, 1.61) |
| >=$100,000 | -29.9  (-65.2, 5.5) | 20.6  (-36.2, 77.4) | -21.6  (-72.6, 29.4) | 1.1  (-2.4, 4.7) | 1.03  (0.92, 1.14) | 1.37  (0.95, 1.98) |
| Parental Education (versus high school) |  |  |  |  |  |  |
| Some college/Assoc | -0.6  (-26.1, 24.9) | **44.7**  **(3.1, 86.2)*** | 36.8  (-0.3, 73.9) | 0.8  (-1.7, 3.4) | 0.97  (0.90, 1.04) | 0.95  (0.75, 1.20) |
| Bachelor’s degree | **50.6**  **(17.1, 84.0)*** | 30.2  (-23.4, 83.9) | **67.9**  **(19.7, 116.1)*** | 1.5  (-1.9, 4.9) | **0.86**  **(0.77, 0.97)*** | **0.56**  **(0.37, 0.84)*** |
| Graduate degree | **34.0**  **(0.2, 67.7)*** | 11.6  (-43.5, 66.7) | 46.6  (-2.5, 95.8) | 0.0  (-3.4, 3.4) | 0.94  (0.84, 1.04) | 0.72  (0.50, 1.05) |
| Parent Martial Status | -8.2  (-27.0, 10.6) | 14.4  (-16.3, 45.0) | 0.6  (-26.8, 27.9) | 0.3  (-1.6, 2.2) | **1.08**  **(1.02, 1.15)*** | 0.96  (0.79, 1.18) |
| Distance home-school | -1.4  (-3.5, 0.6) | -1.3  (-4.6, 2.0) | **-3.3**  **(-6.3, -0.3)*** | 0.0  (-0.2, 0.2) | 1.00  (0.99, 1.01) | 1.01  (0.99, 1.03) |
| Percent time >250m from home | 8.7  (-45.3, 62.7) | 21.6  (-66.6, 109.8) | 57.1  (-21.7, 135.8) | **-5.7**  **(-11.2, -0.3)*** | 0.95  (0.80, 1.14) | 0.65  (0.37, 1.11) |
| School Night (versus non-school night) | **-57.7**  **(-69.0, -46.4)*** | **-49.3**  **(-60.4, -38.2)*** | **-116.1**  **(-128.5, -103.8)*** | 0.0  (-0.8, 0.9) | **1.27**  **(1.19, 1.37)*** | **1.68**  **(1.42, 2.00)*** |
| Month (versus January) |  |  |  |  |  |  |
| February | -4.3  (-46.4, 37.8) | -50.2  (-107.2, 6.8) | **-65.0**  **(-121.7, -8.3)*** | -0.7  (-4.6, 3.2) | 1.04  (0.89, 1.21) | 1.20  (0.68, 2.11) |
| March | -12.3  (-52.8. 28.2) | -49.6  (-106.6, 7.3) | -52.2  (-107.9, 3.5) | -0.9  (-4.8, 2.9) | 1.14  (0.98, 1.31) | 1.16  (0.65, 2.06) |
| April | -8.2  (-48.3, 31.9) | -47.7  (-104.6, 9.2) | -21.1  (-76.4, 34.1) | **-4.0**  **(-7.8, -0.1)*** | 1.08  (0.93, 1.24) | 1.16  (0.66, 2.05) |
| May | -25.5  (-64.1, 13.2) | -49.7  (-104.9, 5.6) | **-57.6**  **(-111.0, -4.2)*** | -2.9  (-6.6, 0.8) | 1.12  (0.98, 1.29) | 1.32  (0.76, 2.30) |
| June | -27.2  (-69.7, 15.3) | 20.8  (-39.9, 81.4) | -6.3  (-64.8, 52.2) | -1.7  (-5.8, 2.3) | 1.05  (0.89, 1.23) | 1.39  (0.74, 2.60) |
| July | **73.1**  **(16.7, 129.6)*** | 8.2  (-67.0, 61.6) | **101.9**  **(27.0, 176.8)*** | 1.3  (-3.8, 6.5) | **0.78**  **(0.64, 0.97)*** | **0.37**  **(0.16, 0.87)*** |
| August | -31.5  (-75.5, 12.6) | -1.8  (-65.2, 61.6) | -3.2  (-63.9, 57.6) | -4.0  (-8.2, 0.2) | **1.21**  **(1.04, 1.42)*** | 1.51  (0.82, 2.80) |
| September | -39.4  (-84.1, 5.4) | -59.8  (-124.4, 4.8) | **-73.4**  **(-135.3, -11.5)*** | -3.1  (-7.4, 1.2) | **1.23**  **(1.05, 1.43)*** | 1.77  (0.90, 3.48) |
| October | -10.8  (-60.3, 38.7) | -64.2  (-138.2, 9.8) | -67.8  (-137.2, 1.6) | -1.9  (-6.7, 2.9) | 1.01  (0.85, 1.20) | 1.31  (0.68, 2.54) |
| November | 14.7  (-35.3, 64.7) | -49.0  (-123.1, 25.1) | 5.7  (-64.0, 75.4) | -4.8  (-9.6,0.0) | 1.00  (0.83, 1.21) | 0.97  (0.49, 1.89) |
| December | -16.9  (-56.9, 23.0) | -21.4  (-75.5, 32.7) | -22.9  (-76.9, 31.2) | -1.3  (-5.0, 2.4) | 1.10  (0.94, 1.30) | 1.49  (0.83, 2.65) |
| Economic deprivation | 0.4  (-13.9, 14.7) | 9.8  (-6.7, 26.2) | -6.0  (-23.3, 11.3) | 0.8 (-0.4, 2.0) | 1.02  (0.97, 1.09) | 0.90  (0.75, 1.07) |
| Social fragmentation | **15.2**  **(3.0, 27.4)*** | -12.0  (-26.1, 2.0) | 13.5  (-1.2, 28.2) | 0.2  (-0.8, 1.2) | **0.94**  **(0.89, 0.99)*** | 0.93  (0.79, 1.10) |
| Tree canopy cover | -4.9  (-16.5, 6.7) | 3.2  (-10.6, 17.0) | -7.9  (-22.0, 6.3) | 0.4  (-0.6, 1.4) | 1.03  (0.98, 1.09) | 0.90  (0.78, 1.05) |
| Walkability | **-11.5**  **(-22.5, -0.4)*** | 7.4  (-5.3, 20.1) | -10.1  (-23.3, 3.2) | 0.2  (-0.7, 1.2) | 1.03  (0.98, 1.09) | 1.00  (0.87, 1.16) |
| Recreational Facilities | 9.8  (-0.4, 20.1) | -5.5  (-17.2, 6.3) | 6.0  (-6.2, 18.2) | -0.5  (-1.3, 0.4) | 0.98  (0.95, 1.02) | 0.95  (0.85, 1.06) |

***p<0.05**

^1^ Models were adjusted for potential confounders including adolescent age, sex, race/ethnicity, household income, parental education, parental marital status, percent of GPS location points located at least 250 meters from home, distance between home and school, school night status, month, and daily activity-space exposure to economic deprivation, social fragmentation, tree canopy cover, walkability, and recreational facilities. Models included participant random intercepts to account for clustering of days within adolescents and used an autoregressive correlation structure. Linear mixed models were used for sleep duration, onset, offset, and efficiency, while modified Poisson regression was used for insufficient and very short sleep.

^2^All neighborhood variables were scaled to reflect the association of an interquartile range difference.

**Supplemental Table 7. Full Regression Model Output- Within-Person Fixed Effects Models^1,2^**

|  | Duration (minutes) – β (95% CI) | Onset (minutes) – β (95% CI) | Offset (minutes) – β (95% CI) | Efficiency (%) - β (95% CI) | Insufficient Sleep (>8 hours) – RR (95% CI) | Very Short Sleep (<6 hours)  – RR (95% CI) |
| --- | --- | --- | --- | --- | --- | --- |
| Crime | 4.5  (-8.2, 17.1) | -6.1  (-19.0, 6.9) | -4.6  (-18.3, 9.2) | 0.3  (-0.7, 1.3) | 1.01  (0.95, 1.08) | 0.95  (0.81, 1.10) |
| Vacant lots | **-34.2**  **(-49.6, -18.7)*** | 18.5  (-0.2, 37.1) | **-20.1**  **(-40.1, -0.1)*** | -0.7  (-1.8, 0.4) | **1.10**  **(1.02, 1.18)*** | **1.28**  **(1.11, 1.48)*** |
| School Night (versus non-school night) | **-55.0**  **(-68.7, -41.2)*** | **-50.8**  **(-65.7, -35.9)*** | **-116.5**  **(-134.3, -98.8)*** | 0.1  (-0.8, 1.0) | **1.26**  **(1.17, 1.35)*** | **1.67**  **(1.40, 1.98)*** |
| Month (versus January) |  |  |  |  |  |  |
| February | -38.9  (-93.9, 16.1) | -21.2  (-62.0, 19.5) | -55.4  (-177.5, 66.7) | **-3.1**  **(-5.7, -0.5)*** | 1.06  (1.00, 1.12) | 1.11  (0.96, 1.28) |
| March | -24.7  (-107.3, 57.9) | -51.4  (-115.9, 13.0) | -56.4  (-187.1, 74.4) | -4.5  (-10.4, 1.3) | 1.04  (0.90, 1.20) | 1.00 (0.60, 1.69) |
| April | 51.0  (-45.6, 147.7) | -65.9  (-138.3, 6.6) | 57.7  (-93.7, 209.1) | -8.4  (-15.3, -1.4) | 0.92  (0.61, 1.37) | **0.37**  **(0.18, 0.77)*** |
| May | 29.0  (-77.7, 135.6) | -78.6  (-175.8, 18.7) | 11.4  (-152.1, 174.8) | -6.6  (-14.8, 1.5) | 0.97  (0.61, 1.54) | **0.42**  **(0.18, 0.97)*** |
| June | 7.0  (-116.1, 130.1) | -3.5  (-122.3, 115.3) | 47.1  (-119.3, 213.6) | -4.2  (-13.3, 4.9) | 0.87  (0.52, 1.45) | 0.40 (0.15, 1.08) |
| July | 135.5  (-51.6, 322.6) | -110.7  (-300.9, 79.5) | 115.4  (-82.2, 313.0) | -2.3  (-12.7, 8.2) | 0.53  (0.156, 1.83) | 0.27  (0.04, 2.00) |
| August | 92.5  (-104.9, 289.8) | -129.4  (-325.0, 66.2) | 36.7  (-169.4, 242.7) | -0.4  (-10.9, 10.2) | 0.56  (0.15, 2.08) | 0.35  (0.02, 7.53) |
| September | 90.3  (-109.0, 289.6) | -179.4  (-376.3, 17.4) | -21.9  (-236.1, 192.3) | 1.1  (-10.0, 12.2) | 0.57  (0.15, 2.12) | 0.34  (0.02, 7.41) |
| October | omitted | omitted | omitted | omitted | omitted | omitted |
| November | omitted | omitted | omitted | omitted | omitted | omitted |
| December | -31.6  (-80.0, 16.8) | **-77.7**  **(-130.0, -25.4)*** | **-134.2**  **(-155.9, -112.5)*** | 2.9  (-4.4, 10.1) | 1.09  (0.68, 1.76) | **1.98**  **(1.15, 3.38)*** |
| Economic deprivation | -17.4  (-38.3, 3.5) | 13.0  (-10.3, 36.4) | -13.3  (-34.8, 8.1) | 0.3  (-1.1, 1.7) | 1.09  (0.98, 1.20) | 1.10  (0.86, 1.40) |
| Social fragmentation | **23.7**  **(3.1, 44.4)*** | -7.9  (-26.0, 10.1) | 18.5  (-5.1, 42.1) | 0.6  (-0.7, 1.8) | **0.91**  **(0.83, 1.00)*** | 0.81  (0.64, 1.03) |
| Tree canopy cover | -11.7  (-29.9, 6.6) | 11.0  (-7.1, 29.1) | -9.9  (-29.8, 9.9) | 1.0  (-0.3, 2.3) | 1.04  (0.94, 1.15) | 1.11  (0.92, 1.34) |
| Walkability | **-19.5**  **(-34.4, -4.5)*** | 10.9  (-3.7, 25.5) | -13.1  (-29.3, 3.2) | 0.5  (-0.7, 1.6) | 1.08  (0.99, 1.18) | **1.18**  **(1.01, 1.39)*** |
| Recreational Facilities | 1.1  (-13.6, 15.8) | -5.3  (-21.7, 11.2) | -2.4  (-15.9, 11.1) | -0.5  (-1.4, 0.5) | 0.98  (0.93, 1.05) | 1.03  (0.88, 1.21) |

***p<0.05**

^1^Within-person associations were estimated using fixed-effects regression to estimate associations of within-person differences in activity space exposures with within-person differences in sleep from night to night. Models were adjusted for time-varying covariates (school night status, month, daily activity-space economic deprivation, social fragmentation, tree canopy cover, walkability, and recreational facilities). Linear models were used for sleep duration, onset, offset, and efficiency, while modified Poisson regression was used for insufficient and very short sleep.

^2^All neighborhood variables were scaled to reflect the association of an interquartile range difference.

**Supplemental Table 8. Sensitivity Analysis Restricting to Adolescents with 5+ Days of Simultaneous GPS and Actigraphy Data (n=121)^1-3^**

|  | Duration (minutes) – β (95% CI) | Onset (minutes) – β (95% CI) | Offset (minutes) – β (95% CI) | Efficiency (%) - β (95% CI) | Insufficient Sleep (>8 hours) – RR (95% CI) | Very Short Sleep (<6 hours)  – RR (95% CI) |
| --- | --- | --- | --- | --- | --- | --- |
| Crime |  |  |  |  |  |  |
| In Home Buffer | **-16.4**  **(-27.5, -5.3)*** | 5.0  (-14.6, 24.5) | -17.3  (-35.2, 0.6) | 0.1  (-1.2, 1.4) | **1.04**  **(1.01, 1.07)*** | **1.18**  **(1.07, 1.30)*** |
| In School Buffer | **-14.7**  **(-22.3, -7.1)** | **13.2**  **(0.3, 26.1)*** | -3.1  (-15.4, 9.2) | -0.4  (-1.3, 0.4) | **1.04**  **(1.02, 1.06)*** | **1.10**  **(1.03, 1.17)*** |
| In Daily Activity Space | -8.5  (-18.4, 1.5) | -6.6  (-18.2, 4.9) | **-13.7**  **(-25.9, -1.5)*** | 0.2  (-0.6, 1.1) | **1.04**  **(1.00, 1.08)*** | **1.11**  **(1.01, 1.23)*** |
| Within-Person | 5.1  (-7.7, 18.0) | -7.6  (-20.8, 5.7) | -5.9  (-19.9, 8.1) | 0.4  (-0.7, 1.4) | 1.01  (0.95, 1.07) | 0.95  (0.81, 1.11) |
| Vacant Lots |  |  |  |  |  |  |
| In Home Buffer | -2.4  (-11.6, 6.8) | 3.9  (-12.1, 19.8) | 3.4  (-11.3, 18.1) | -0.1  (-1.2, 0.9) | **1.02**  **(1.00, 1.05)*** | 0.99  (0.89, 1.09) |
| In School Buffer | -9.1  (-18.7, 0.5) | 6.9  (-9.7, 23.5) | -7.6  (-23.4, 8.1) | 0.4  (-0.7, 1.5) | 1.03  (1.00, 1.06) | 1.00  (0.91, 1.11) |
| In Daily Activity Space | **-15.3**  **(-23.8, -6.9)*** | **12.5**  **(1.3, 23.6)*** | -7.4  (-18.8, 4.0) | -0.7  (-1.5, 0.1) | **1.05**  **(1.02, 1.07)*** | 1.05  (0.94, 1.16) |
| Within-Person | **-34.0**  **(-50.4, -17.7)*** | 16.2  (-3.0, 35.4) | **-22.0**  **(-42.9, -1.2)*** | -0.8  (-2.0, 0.5) | **1.10**  **(1.02, 1.19)*** | **1.27**  **(1.10, 1.47)*** |

***p<0.05**

^1^Results are from 4 sets of models. Model 1 examined associations for crime and vacant lot exposure within a 660 ft buffer (approximately 1 city block) around participants home address (person-level exposure). Model 2 examined associations for crime and vacant lot exposure within a 660ft buffer around participants’ school address (person-level exposure). Model 3 examined associations for daily activity-space crime and vacant lot exposures (person-day-level exposure). Model 4 examined associations of within-person differences in daily activity space crime and vacant lot exposures (e.g., differences in sleep on days when adolescents had higher exposure than their own average level).

^2^Models were adjusted for potential confounders including adolescent age, sex, race/ethnicity, household income, parental education, parental marital status, percent of GPS location points located at least 250 meters from home, distance between home and school, school night status, month, and daily activity-space exposure to economic deprivation, social fragmentation, tree canopy cover, walkability, and recreational facilities. Models included participant random intercepts to account for clustering of days within adolescents and used an autoregressive correlation structure. Linear mixed models were used for sleep duration, onset, offset, and efficiency, while modified Poisson regression was used for insufficient and very short sleep.

^3^Within-person associations were estimated using fixed-effects regression to estimate associations of within-person differences in activity space exposures and within-person differences in sleep from night to night. Models were adjusted for time-varying covariates (school night status, month, daily activity-space economic deprivation, social fragmentation, tree canopy cover, walkability, and recreational facilities). Linear models were used for sleep duration, onset, offset, and efficiency, while modified Poisson regression was used for insufficient and very short sleep.

^4^All crime and vacant lot variables were scaled to reflect the association of an interquartile range difference.
